# Comprehensive Analysis of Five *Phyllostachys edulis* *SQUA*-like Genes and Their Potential Functions in Flower Development

**DOI:** 10.3390/ijms221910868

**Published:** 2021-10-08

**Authors:** Yuting Zhang, Junhong Zhang, Minyan Song, Xinchun Lin, Zaikang Tong, Mingquan Ding

**Affiliations:** 1 State Key Laboratory of Subtropical Silviculture, Zhejiang A&F University, Lin’an, Hangzhou 311300, China; zhangyt@zafu.edu.cn (Y.Z.); zhangjunhong@zafu.edu.cn (J.Z.); sennsong@163.com (M.S.); lxc@zafu.edu.cn (X.L.); 2 The Key Laboratory for Quality Improvement of Agricultural Products of Zhejiang Province, College of Advanced Agricultural Sciences, Zhejiang A&F University, Lin’an, Hangzhou 311300, China

**Keywords:** SQUA-like genes, *Phyllostachys edulis*, floral development, protein–protein interaction (PPI), ectopic expression, early flowering

## Abstract

Bamboo is one of the most important non-timber forest resources worldwide. It has considerable economic value and unique flowering characteristics. The long juvenile phase in bamboo and unpredictable flowering time limit breeding and genetic improvement and seriously affect the productivity and application of bamboo forests. Members of *SQUA*-like subfamily genes play an essential role in controlling flowering time and floral organ identity. A comprehensive study was conducted to explain the functions of five *SQUA*-like subfamily genes in *Phyllostachys edulis.* Expression analysis revealed that all *PeSQUAs* have higher transcript levels in the reproductive period than in the juvenile phase. However, *PeSQUAs* showed divergent expression patterns during inflorescence development. The protein–protein interaction (PPI) patterns among PeSQUAs and other MADS-box members were analyzed by yeast two-hybrid (Y2H) experiments. Consistent with amino acid sequence similarity and phylogenetic analysis, the PPI patterns clustered into two groups. PeMADS2, 13, and 41 interacted with multiple PeMADS proteins, whereas PeMADS3 and 28 hardly interacted with other proteins. Based on our results, PeSQUA might possess different functions by forming protein complexes with other MADS-box proteins at different flowering stages. Furthermore, we chose *PeMADS2* for functional analysis. Ectopic expression of *PeMADS2* in *Arabidopsis* and rice caused early flowering, and abnormal phenotype was observed in transgenic *Arabidopsis* lines. RNA-seq analysis indicated that *PeMADS2* integrated multiple pathways regulating floral transition to trigger early flowering time in rice. This function might be due to the interaction between PeMADS2 and homologous in rice. Therefore, we concluded that the five *SQUA*-like genes showed functional conservation and divergence based on sequence differences and were involved in floral transitions by forming protein complexes in *P. edulis*. The MADS-box protein complex model obtained in the current study will provide crucial insights into the molecular mechanisms of bamboo’s unique flowering characteristics.

## 1. Introduction

Bamboos are important members of the subfamily Bambusoideae and the family Poaceae, which are important timber, fiber, and food products worldwide [1]. Unlike other members of Poaceae, bamboo has unique flowering features and an unpredictable juvenile phase [2]. For instance, wood bamboo retains vegetative growth for a very long time (13–120 years) and usually dies after seed production [1]. The flowering incidence may be restricted to a few plants of a population, i.e., so-called sporadic flowering, whereas synchronous flowering may happen across populations in a community in which all plants flower and die within the same year [3]. The long juvenile phase in bamboo and unpredictable flowering time possibly limit breeding and genetic improvement and cause economic losses. Consequently, studying the mechanism of bamboo flowering has great scientific importance and economic value. Although many research groups tried to uncover the cause of bamboo flowering by genomes and transcriptomes [4,5,6], the molecular aspects of bamboo flowering remain unclear.

*Phyllostachys edulis* (Moso bamboo) genome and the transcriptomes of different bamboo species have been sequenced to characterize genes related to bamboo flowering; most of the cloned genes belonged to the MADS-box gene family [2,5,7,8]. For instance, 16 *BeMADS* genes were obtained from the flowering transcripts of *Bambusa edulis*, among which BeMADS1 could undergo co-transformation with other cytosol BeMADS proteins in the nucleus to function as transcription factors (TFs) [8]. *PvSOC1* and *PvMADS56* from *P. violascens* can significantly promote flowering in overexpression transgenic *Arabidopsis thaliana* or *Oryza* [9,10]. BoMADS50 could interact with SQUAMOSA (SQUA)-like proteins and positively promote flowering in *B. oldhamii* [6]. Based on previous results from our laboratory, 42 full-length *P. edulis* MADS-box members were identified, and the expression analysis revealed that several *PeMADSs* play roles in modulating flowering in Moso bamboo [11]. Ectopic expression of one of the candidate genes, namely, *PeMADS5*, could promote flowering in *A. thaliana* [11].

In plants, MADS-box gene family members reportedly play important roles in many aspects of development [12,13,14], particularly the regulation of reproductive development, including the flowering time, establishing floral organ identity, fruit ripening, seed pigmentation, and embryo development [15,16,17,18]. Members from the *SQUA*-like subfamily, also named as *APETALA1/FRUITFULL* (*AP1/FUL*), participate in floral meristem identity, floral organ specification, and flowering regulation [16]. There are four members of the *Arabidopsis SQUA* subfamily in which the roles of *AP1*, *CAULIFLOWER* (CAL), and FUL are crucial for the control of floral organ development and flowering time [19]. *AP1* belongs to the A-class gene in the floral organ ABC model, and it is involved in the identification of sepal and petal development [20]. Meanwhile, *AP1* and *CAL* share overlapping roles in floral meristem (FM) identity and determination [21]. *FUL* has an important role in *Arabidopsis* carpel and fruit development and is functionally redundant with *AP1* and *CAL* in controlling inflorescence architecture [21,22]. The function of *SQUA*-like genes that control floral transition is somewhat conserved between eudicots and grass [23]. In rice, *SQUA*-like genes have redundant roles in controlling flowering time, and the ectopic expression of *OsMADS14*, *OsMADS15*, or *OsMADS18* results in early flowering and dwarf habit [24,25]. In winter wheat and barley varieties, overexpression of the *SQUA*-like gene (*VERNALIZATION 1*) could promote flowering in response to vernalization [26,27]. Two bamboo *SQUA*-like subfamily genes, *PpMADS1* and *PpMADS2*, were identified from *P. praecox*, and overexpression of these two genes significantly promotes early flowering in *Arabidopsis* [28].

Several studies support the important roles of *SQUA*-like genes in floral transition and inflorescence architecture. However, few studies investigated the regulatory roles of *SQUA*-like genes in *P. edulis*, which is the most commonly used species in China and is the only bamboo with whole-genome information released [18]. In this study, we identified five *SQUA*-like members in *P. edulis*. The expression patterns of *PeMADSs* at different flowering stages were comprehensively studied. Yeast two-hybrid (Y2H) assay showed that some *SQUA*-like members have extensive interactions with other MADS-box subfamilies. The Y2H assay showed that PeMADS2 has the highest number (11) of interaction proteins among the five PeSUQAs subgroup members, and *PeAMDS2* was highly expressed during inflorescence initiation. Thus, we prioritize *PeMADS2* for further functional study. The ectopic expression of *PeMADS2* in *Arabidopsis* caused early flowering and abnormal phenotype in leaves and inflorescence. Furthermore, overexpression of *PeMADS2* in rice also triggered early flowering time, and RNA-seq analysis indicated that *PeMADS2* integrated multiple flower signaling pathways in transgenic plants. Our data provided the spatial expression analysis and protein–protein interaction (PPI) of *SQUA*-like genes with other bamboo MADS-box members. Our findings present new perspectives that contribute to the function of *PeMADS2* in regulating the floral transition in transgenic *Arabidopsis* and rice.

## 2. Results

### 2.1. The Bioinformatics Analysis of Five SQUA-like Members in P. edulis

**Figure 1:**
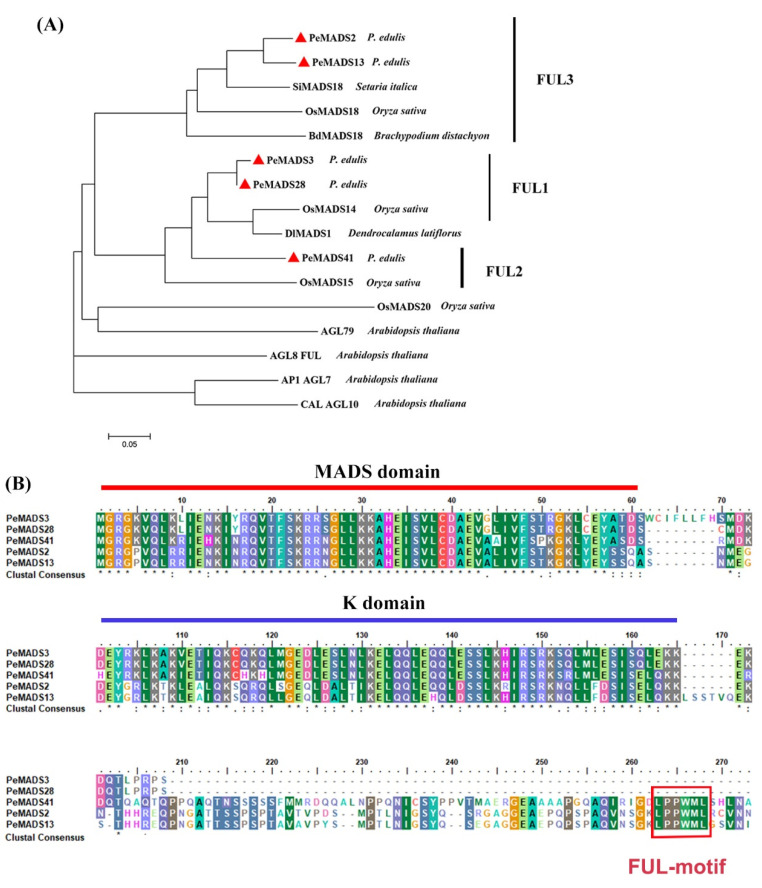
Sequence analyses of PeSQUA-like. (**A**) A phylogenetic tree of Moso bamboo PeSQUA-like and SQUA-like proteins from other species. The phylogenetic tree was constructed with the neighbor-joining (NJ) method and evaluated by bootstrap analysis using MEGA soft (version 7.0). Sixteen SQUA-like proteins were used: four from *A. thaliana* (AGL7, AGL9, AGL10, and AGL79), four from rice (OsMADS14, OsMADS15, OsMADS18 and OsMADS20), one from *Brachypodium distachyon* (BdMADS18), one from *Setaria italic* (SiMADS18), and one from *D. latiflorus* (DlMADS1). *P. edulis* SQUA-like proteins are marked by red triangles. (**B**) Alignment of AA sequences of *P. edulis* SQUA-like proteins. The lined regions represent the conserved MADS domain and K domain. The red boxed region in the C-terminal represents the FUL-like motif. Residues that are identical in all sequences in the alignment are marked with “*” in the bottom row, conserved and semi-conserved substitutions with “:” and “.” respectively.

Five *SQUA*-like genes, namely, *PeMADS2, PeMADS3, PeMADS13, PeMADS28,* and *PeMADS41*, were obtained from our previous study [11]. The phylogenetic tree of SQUA-like proteins showed that these five *P. edulis SQUA*-like genes are clustered within monocot species (*Oryza sativa*, *Brachypodium distachyon*, *Setaria italica*, and *D. latiflorus*) and are separate from *Arabidopsis* (Figure 1A). PeMADS2 and PeMADS13 were clustered in one branch with rice OsMADS18, whereas PeMADS3 and PeMADS28 were gathered in the other branch with rice OsMADS14. However, PeMADS41 showed less homology with the other four genes. The alignment indicated that all five bamboo SQUA-like proteins contained a well-conserved MADS-domain in the N-terminus, followed by the less conserved K (keratin-like) domain, and a divergent C-terminal region (Figure 1B). Furthermore, a conserved FUL-like (LPPWML) motif was identified in the C-terminal region of PeMADS2, PeMADS13, and PeMADS41, which was highly conserved in most monocot SQUA-like genes [29]. However, this motif was absent in PeMADS3 and PeMADS28 due to the truncated C terminus (Figure 1B).

### 2.2. Expression Analysis of PeSQUA-like Genes during Different Flowering Stages

**Figure 2:**
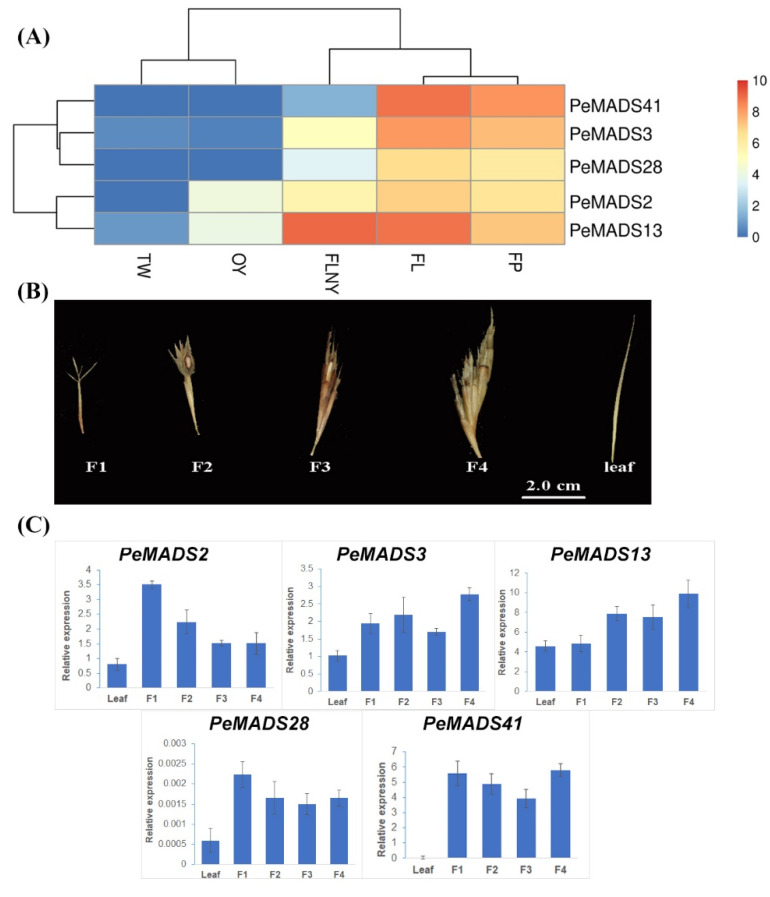
The expression pattern of *PeSQUA*-like genes. (**A**) The expression levels of *PeSQUAs* during the transition from vegetative to reproductive growth. TW: leaves from 3-week-old seedlings, OY: leaves from 1-year-old plants, FLNY: leaves from plants that will flower in the next year, FL: leaves from flowering plants, and FP: flower florets. (**B**) The bamboo inflorescence development was divided into four stages: the first floral bud formation (F1), the initial stage of inflorescence development (F2), maturation of inflorescence (F3), and anthesis (F4). The leaf tissues (leaf) from the non-flowering plant. (**C**) qPCR analysis of *PeSQUAs* at different flowering stages. *NTB* or *TIP41* was used as a reference gene; mean ± SD of three biological replicates is presented.

To better elucidate the roles of *PeSQUA*-like genes in the transition from vegetative growth to flowering, the expression levels were analyzed from online transcriptomes [30]. The transcriptomes include leaf samples from different developmental stages, as follows: 3-week-old seedlings (TW); 1-year-old plants (OY); plants that will flower in the next year (FLNY); and flowering plants (FL), and flower florets (FP) samples from the flowering stage. The *PeSQUA*-like genes were predominantly detected in leaves (FL) and florets (FP) from flowering plants and were moderately expressed in plants ready to flower (FLNY). However, they were poorly expressed in juvenile plants (TW and OY) (Figure 2A). Based on the expression patterns, the *PeSQUAs* were divided into two groups, which were similar to the homology tree (Figure 1A). Besides the flowering tissues, the transcripts of *PeMADS2* and *PeMADS13* were both high in the plants at the reproductive transition stage (FLNY).

*PeSQUA*-like gene expression levels at different flowering developmental stages were also tested by qPCR. Bamboo inflorescence development (Figure 2B) had four stages, namely, the first floral bud formation (F1), the initial stage of inflorescence development (F2), maturation of inflorescence (F3), and anthesis (F4). The leaf tissues (leaf) from the non-flowering plant under the same growth environment as the flowering plants were used as the control. The transcripts of *PeSQUAs* were highly expressed in floral samples and less expressed in leaves. The expression of *PeMADS2* exhibited a peak expression at the initial stage (F1) and decreased during inflorescence development, whereas *PeAMDS3* and *PeMADS13* showed an upregulated trend. The expression levels of *PeMADS28* and *PeMADS41* showed a mild change at different floral development stages.

### 2.3. PPI between PeSQUA-like Members and Other MADS-Box Proteins in P. edulis

**Figure 3:**
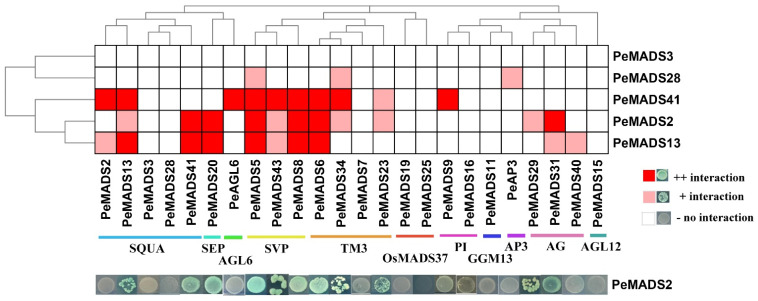
Protein–protein interactions (PPI) among PeSQUAs and PeMADSs. A yeast two-hybrid assay was performed to verify the interaction among PeSQUAs and other MADS-box proteins. The result was displayed by the phylogenetic tree of the bamboo MADS-box gene family. The dark red represents a strong reaction, light red represents a mild reaction, and gray represents no reaction.

To investigate the interaction patterns of *P. edulis SQUA*-like members, a comprehensive Y2H assay was performed to clarify the protein interactions among bamboo MADS-box proteins (Figure 3). The five BD baits of PeSQUAs showed no auto-activation or toxicity (Figure 1). Among five genes, PeMADS2 had the highest number of interaction relationships with other MADS-box proteins, followed by PeMADS13 and PeMADS41. PeMADS13 could form a homologous dimer, whereas the other four PeSQUAs could not. Based on the PPI results, the PeSQUAs could be classified into two groups, thereby showing the variable PPI patterns. PeMADS2, 13, and 41 had an extensive interaction network among eight MADS-box subfamilies, whereas the PeMADS28 and PeMADS3 showed fewer or no interactions with other PeMADSs. Most of the interaction relationships were among SQUA-, SVP-, TM3-, and AG-like subgroups. The SVP-like proteins showed the strongest correlation with SQUA-like proteins, specifically with PeMADS2, 13, and 41.

### 2.4. Ectopic Expression of PeMADS2 Accelerates Arabidopsis Flowering

**Figure 4:**
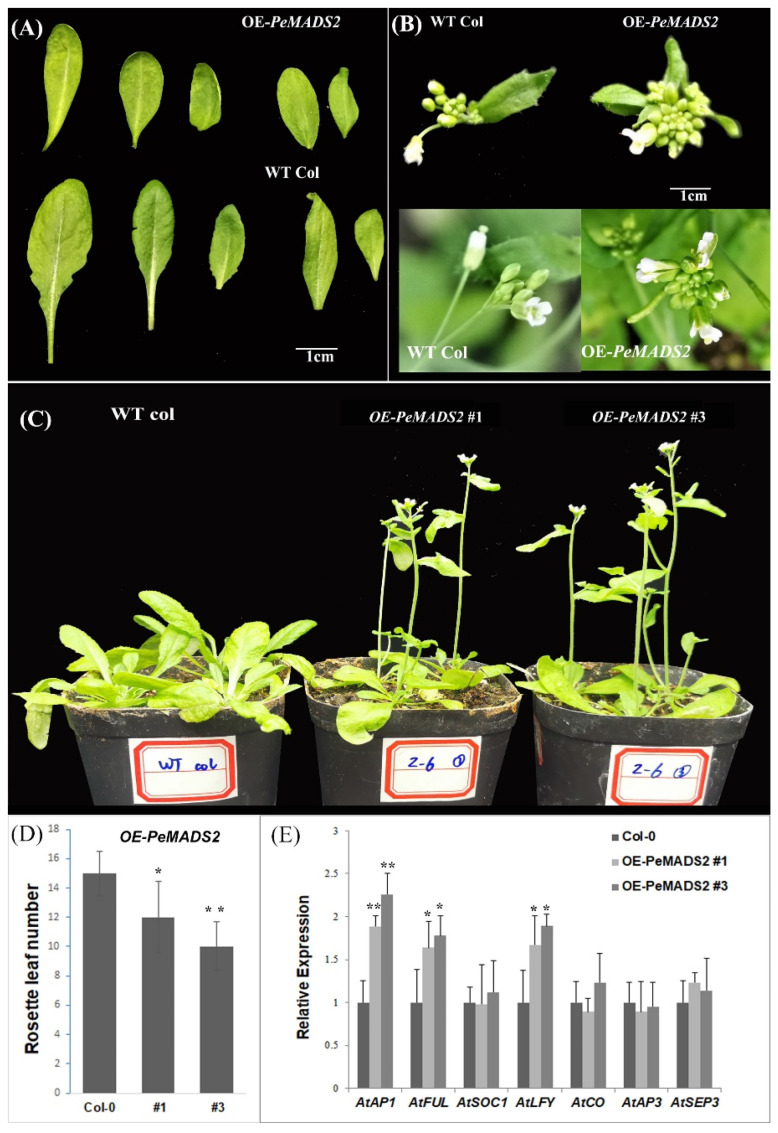
The early flowering phenotype of OE-*PeMADS2* in *Arabidopsis*. (**A**)The leaves of OE-*PeMADS2* transgenic and WT *Arabidopsis* plants. (**B**) The floral organs of OE-*PeMADS2* transgenic *Arabidopsis* and wild-type plants. (**C**) Phenotypes of overexpressing *PeMADS2* transgenic lines (OE-*PeMADS2*#1, #3) and wild-type (WT) plants as control under LD conditions. (**D**) Flowering time was scored as the number of rosette leaves under LD conditions. (**E**) Transcription levels of *AP1*, *FUL*, *LFY*, *CO*, *AP3*, *SOC1*, and *SEP3* in WT and transgenic plants. *Arabidopsis* Actin or *TIP41* was used as the internal reference gene. Mean ± SD of three biological replicates is presented. Asterisks indicate a significant difference between transgenic and WT plants (* *p* ≤ 0.05, ** *p* ≤ 0.01, *t*’s test).

To explore the roles of bamboo *SQUA-like* genes in the regulation of flowering, we transformed *PeAMDS2* into *Arabidopsis*. *PeAMDS2* was highly expressed during inflorescence initiation and had the highest PPI potential. The ectopic expression of *PeAMDS2* in *Arabidopsis* resulted in an early flowering phenotype under long-day (LD) conditions (Figure 4C), and the phenotypic variations were observed in leaves and floral organs (Figure 4A,B). The rosette and cauline leaves of OE-*PeMADS2* plants were curled and smaller than those of the wild-type (WT) plants (Figure 4A). The inflorescences were densely clustered in the transgenic plants (Figure 4B). The flowering time was approximately 6–8 days earlier than WT plants, and the number of rosette leaves of OE-*PeMADS2* plants was significantly fewer than that of WT *Arabidopsis* (Figure 4D). We further investigated the expression levels of important genes involved in flowering time and flower organ development. The expression levels of *AtAP1*, *AtFUL,* and *LEAFY* (*AtLFY*) were significantly upregulated, whereas the expression levels of the other four genes were insignificantly different between transgenic and WT plants (Figure 4E). *PeMADS2* might regulate transgenic plant flowering by controlling the expression of its homology (*AtAP1* and *AtFUL*) and *AtLFY.*

### 2.5. Ectopic Expression of PeMADS2 Promotes Rice Flowering

**Figure 5:**
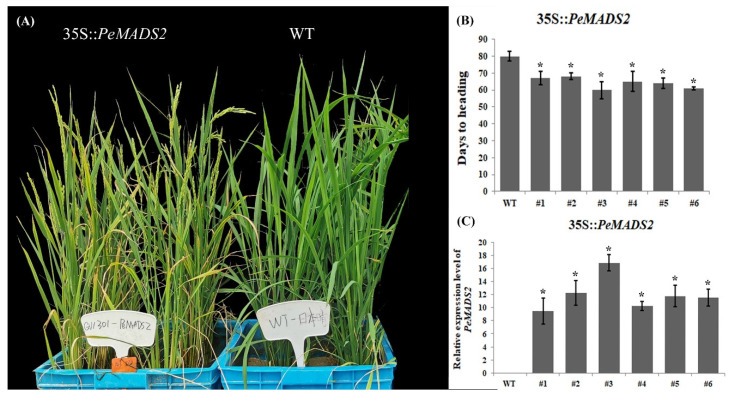
Ectopic expression of *PeMADS2* in *Oryza*. (**A**) Representative picture of WT and ectopic expression lines of *PeMADS2* after heading. (**B**) Days to the heading of T3 homozygous transgenic plants. (**C**) Relative expression levels of *PeMADS2* in transgenic rice plants and WT. Asterisks indicate a significant difference between transgenic and WT plants (* *p* ≤ 0.05, *t*’s test).

To further examine the function of hub genes, *PeMADS2* was transformed into *O. sativa* (Dongjing), a member of the same grass family as bamboo. Twenty-four T_1_ OE-*PeMADS2* transgenic rice plants were obtained. We analyzed the heading time and found that OE-*PeMADS2* transgenic rice plants had a significantly early flowering phenotype (Figure 5A). In transgenic rice, the average relative expression level of *PeMADS2* was ~12.05 ± 2.57 (Figure 5C). The heading time of transgenic plants (Figure 5B) was ~64.17 ± 3.18 d, which was 16 d earlier than that of the WT lines (80.62 ± 1.02 d). Furthermore, the highest level of *PeMADS2* was detected in OE-*PeMADS2* line #3, which showed the earliest heading, thereby indicating a negative association between the heading time and the expression level of *PeMADS2*.

### 2.6. Analysis of Differential Gene Expression in OE-PeMADS2 Transgenic Rice

**Figure 6:**
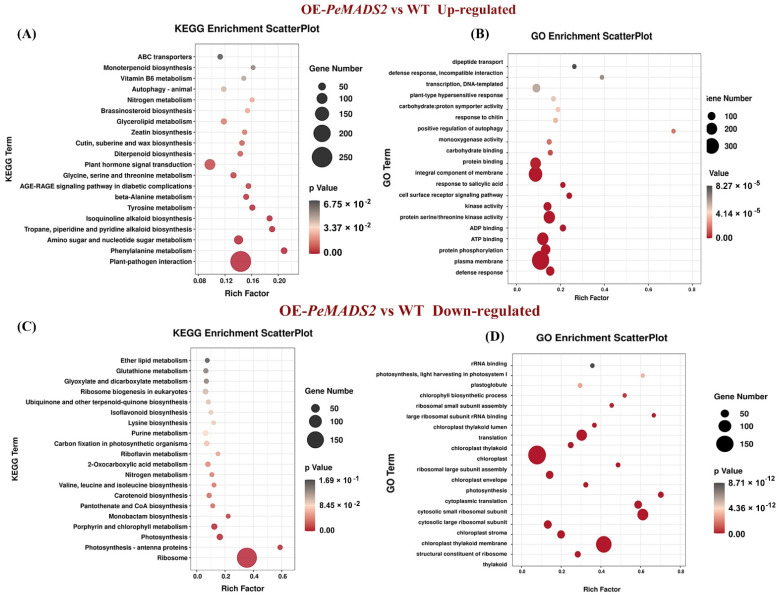
Analysis of RNA-seq data of OE-*PeMADS2* lines and wild-type rice plants. (**A**,**B**): GO and KEGG enrichment analysis of DEGs that were upregulated in OE-*PeMADS2* plants. (**C**,**D**): GO and KEGG enrichment analysis of DEGs that were downregulated in OE-*PeMADS2* plants. Low q-values are shown in the dark grey circle, and high q-values are shown in the red circle. The size of a circle represents DEG number.

To explore the early heading mechanism in transgenic rice, the RNA-seq analyses of OE-*PeMADS2* (line #3) and WT lines were performed. We pooled the short reads and aligned them to the Nipponbare reference genome to identify the transcripts. In total, we obtained 145,947,014 and 136,115,288 reads with three biological repeats from OE-*PeMADS2* transgenic rice and wild-type rice libraries, respectively. The mapping rate for each library was above 95%. We obtained 39,333,958 (85.74%) and 38,097,230 (86.52%) uniquely mapped reads for further analysis (Supplemental Table S4). Among the 42,004 expressed unigenes, 27,213 have been GO annotated, and 14,606 have been KEGG annotated. Compared with WT lines, 3146 genes were differentially expressed, including 2021 that were upregulated and 1125 that were downregulated in OE-*PeMADS2* lines. KEGG analysis indicated that the genes for plant–pathogen interaction and plant hormone signal transduction were significantly upregulated in transgenic lines (Figure 6A). In contrast, genes for ribosome and photosynthesis were significantly downregulated (Figure 6C). GO enrichment showed that genes upregulated in OE-*PeMADS2* lines were enriched in defense response, plasma membrane, integral component of membrane, and ATP binding (Figure 6B). Differentially expressed genes (DEGs) in OE-*PeMADS2* plants were enriched for functions related to floral development with overrepresented gene ontology (GO) terms such as “regulation of timing of the transition from vegetative to reproductive phase” (GO:0048510) and “flower development” (GO:0009908). Genes associated with plant hormone signal transduction, such as the salicylic acid- (GO:0009751, GO:0071446, and GO:2000031) and abscisic acid-activated signaling pathways (GO:0009738, GO:0010427), were overrepresented in OE-*PeMADS2* (Supplemental Table S5). In contrast, downregulated GO terms were involved in the structural constituents of ribosome, chloroplast, and translation (Figure 6D, Supplemental Table S6).

**Figure 7:**
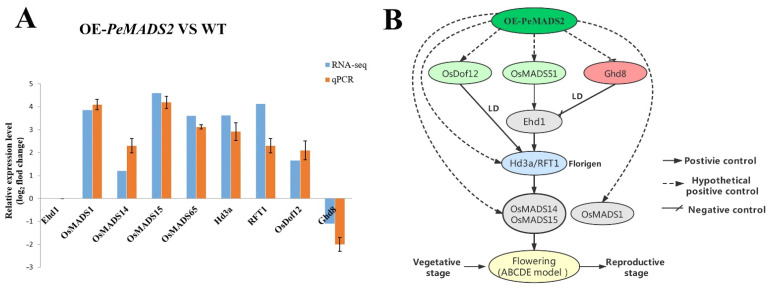
Proposed working models of OE-*PeMADS2* to induce the early flowering in transgenic rice. (**A**) Relative expression pattern analysis of rice floral-related genes by qRT–PCR analysis to validate RNA-seq data. Rice *Ubq* or *EP* were used as reference genes; mean ± SD of three biological replicates is presented. (**B**) The model of the early flowering signal pathway in OE-*PeMADS2* transgenic rice. Arrows with solid lines indicate positive regulators of flowering, arrows with lines indicate negative regulators of flowering, and arrows with dotted lines indicate hypothetical positive control of flowering.

Thirty-seven genes involved in rice floral regulatory pathways were identified for further analysis based on transcription data (Supplemental Table S7). DEGs with a minimum fold change 1 (Log_2_ converted) and a q-value of <0.05 were identified and extracted (Table 1). *OsMADS1*, *OsMADS15*, *OsMADS14*, *OsMADS51* (*OsMADS65*), *DNA-BINDING WITH ONE FINGER* (*OsDof12*), *HEADING DATE 3a* (*Hd3a*), and *RICE FLOWERING LOCUS T 1* (*RFT1*) were all upregulated in the transgenic lines compared with WT. The expression levels of GRAIN NUMBER, PLANT HEIGHT, AND HEADING DATE 8 (*Ghd8*) were downregulated. The transcriptome results were further validated by qPCR analysis (Figure 7A), which showed similar expression trends with RNA-seq data.

## 3. Discussion

### 3.1. Gene Expression Patterns and PPI Patterns Are Divergent among PeSQUA-like Genes

*SQUA*-like MADS-box genes are typical A-class floral organ identity genes that have essential roles in modulating floral transition and organ development [23]. Previous studies often focus on the extensive evolution and development studies of *SQUA*-like genes in rice and wheat [20,26], whereas relatively little information on their functions in bamboo is available. In the present study, five *SQUA*-like MADS-box genes were characterized from *P. edulis,* and these were grouped into two clusters, depending on the existence of the FUL-like motif in the C-terminal region (Figure 1). Sequence structures that have similar features are likely to share a relatively closer evolutionary relationship, especially if the features appear in a non-conserved region [31]. In our study, this FUL-like motif seems to be important for the function of bamboo *SQUA*-like proteins. The expression profiles revealed that *PeSQUAs* from the same lineage share a similar expression pattern, which is consistent with phylogenetic studies (Figure 2). Although the five *PeSQUAs* genes were highly expressed in leaves and florets of flowering plants, *PeMADS2* and *PeMADS13* have relatively higher transcript levels in the reproductive transformation period, thereby suggesting that *PeMADS2* and *PeMADS13* are involved in floral transition modulation. The conserved FUL-like motif consisting of hydrophobic amino acids might be crucial in PPIs [32]. Consistently, cluster I, which includes PeMADS2, 13, and 41 that harbor FUL-like motifs, could interact with multiple PeMADS proteins, whereas PeMADS3 and 28 have no FUL-like motif and hardly interact with other proteins (Figure 3). Although the importance of the FUL-like motif to protein function is unclear in bamboo, the differences of these sequences in the C-terminal region might explain the divergences in interacting and expression patterns of *PeSQUAs* [32]. Thus, PeMADS2, 13, and 41 might play more important roles than PeMADS3 and 28 in the floral development of *P. edulis*.

### 3.2. PPI Patterns of PeSQUAs Alternating from Vegetative to Reproductive Growth

**Figure 8:**
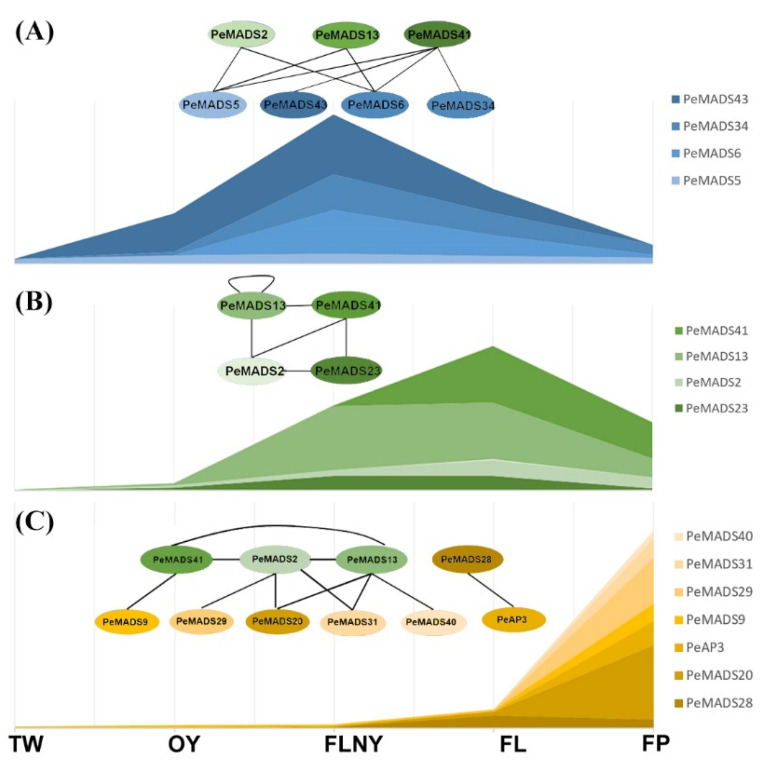
A hypothesis of the molecular model of PeSQUAs in flower development. The stacked graphs represent bamboo MADS-box gene expression levels. The circle represents MADS-box proteins. The solid lines between different proteins indicate the PPI validated by Y2H. The model in the FLNY stage (**A**), the model in the FL (**B**), and the model in the FP (**C**). Each oval represents a different PeMADS protein.

The change from vegetative to reproductive growth involves many genes. Understanding the PPI networks of the master floral developmental regulator is necessary to obtain the full picture of the phase transition in *P. edulis*. Thus, we proposed a hypothetical molecular model based on the *PeSQUAs* expression patterns and the PPI network during reproductive phase transitions (Figure 8). *SQUA*-like genes play key roles in several important developmental processes, especially in flower development [33]. In rice, the interaction of OsMADS18, OsMADS14, and OsMADS15 with other MADS proteins suggested that monocot *SQUA*-like genes accomplish a general role in floral transition and floral meristem identity by coordinating their functions [34]. Consistently, bamboo *SQUA*-like genes show high expression levels at the reproductive stage, and the potential interactors, i.e., SVP-like genes (*PeMADS5* and *43*) and TM3-like genes (*PeMADS6* and *34*), showed higher accumulation during the FLNY stage (Figure 8A). This finding suggested that the formation of dimers between *SQUA*-like and SVP-like genes or TM3-like genes triggers the initial stages of flower development. The expression levels of bamboo SQUA-like genes (PeMADS2, 13, 41, and 28) were higher in the leaves (FL/FLNY) than the floral organs (FP) at reproductive stages (Figure 8B). Rice *SQUA*-like genes (*OsMADS14* and *15*) were largely detected in the floral meristem and organs [35], whereas *OsMADS18* was expressed in most tissues and increased during rice reproduction [25,34]. The PPI relationship among the SQUA-like subfamily was similar to rice in that the members could interact with one another [23,34]. Our results indicated that PeMADSs played important roles in the process of floral transition and morphological architecture due to the formation of PeSQUA homo- or heterodimer. In the floral organ (Figure 8C), PeSQUA proteins might interact with SEP-(PeMADS20), PI-(PeMADS9), AG-(PeMADS29, 31, and 40), and AP3-like (PeAP3) subfamily members; these genes are required for floral development in grasses corresponding to A-, B-, and C- classes of the ABC model [36]. These MADS protein complexes may play certain roles in floral meristem identity and inflorescence development. Based on our results, PeSQUAs might possess different functions by forming complicated protein complexes with other MADS-box proteins.

### 3.3. PeMADS2 Acts as a Flowering Promoter in Transgenic Arabidopsis

Several studies have shown that the increased expression of *SQUA*-like genes in monocots is correlated with the floral transition and that the ectopic expression of all rice *SQUA* subfamily members results in early flowering and dwarfism [24,25]. This was in line with this study that ectopic expression of *PeMADS2* had an early flowering phenotype in *Arabidopsis*. Ectopic expression of *PeMADS2* led to the up-regulation of *AtAP1*, *AtFUL*, and *AtLFY* genes, which are associated with the flowering regulation (Figure 4E). These results suggest that the functions of *PeMADS2* might be dependent on its homologs, *AP1* and *FUL*, in Arabidopsis. However, the underlying mechanism mediated by *PeMADS2* in Arabidopsis needs further investigation. The ectopic expression of *TaVRN1*, a *SQUA*-like member in wheat, caused the overexpression of *Arabidopsis AP1*, which resulted from the binding of TaVRN1 to CArG-box present on the *AP1* promoter [37]. CArG-box was the binding site for the MADS domain transcription factors [38]. Therefore, in silico identification of the CArG-box motif was performed in the 3000 bp upstream of *AtAP1*, *AtFUL*, and *AtLFY* (Supplemental Table S8). At least two CArG-boxes were presented in the promoter regions of these three genes. According to the result, we speculated that PeMADS2 might promote transgenic *Arabidopsis* flowering by physically binding *AtAP1*, *AtFUL*, and *AtLFY’s* promoters to trigger the up-regulation of the genes’ expression, however, further “in vitro” and “in vivo” experimental verifications should be included in the future.

The ectopic expression of *PeMADS2* in *Arabidopsis* and rice both showed early flower phenotype and caused up-regulation of flowering regulation genes (Figure 4E and Table 1). In both *Arabidopsis* and rice, the ectopic expression of *PeMADS2* triggered the accumulation of its corresponding homologous genes. The upregulated *Arabidopsis AtAP1* and *AtFUL* genes and rice *OsMADS14* and *OsMADS15* genes, belonged to the *SQUA*-like subgroup. Furthermore, the key flowering regulators, *AtLFY* in *Arabidopsis* and *Hd3a*/*RFT1* in rice, were both significantly upregulated in the OE-*PeMADS2* transgenic plants. The *Arabidopsis LFY* plays a key role in reproductive transitions via the regulation of floral homeotic MADS-box transcription factors [39]. The rice *RFT1* and *Hd3a* are thought to encode the mobile flowering signal (florigens) proteins and promote floral transition [40]. However, the number of flowering-related DEGs is greater in transgenic rice than that in *Arabidopsis*, which might be due to the closer biological relationship between rice and Moso bamboo.

### 3.4. Ectopic Expression of PeMADS2 Triggered Multiple Flower Signaling Pathways to Induce Early Flowering in Rice

Genetic approaches in Moso bamboo are not feasible for now, and advanced experiments are performed in the most related model species and rice. The ectopic expression of *PeMADS2* had an early flowering phenotype; the heading date was approximately 15 days earlier than in WT (Figure 5A). Based on transcriptomic data and qPCR, we proposed the working model of *PeMADS2* to induce early flowering (Figure 7B). One of the regulation modules triggered is the *OsMADS51/65*-*Hd3a/RFT1*-*OsMADS14/15* pathway. *OsMADS51/OsMADS65*, a type I MADS-box gene that can promote flowering through the *Ehd1*-dependent pathway via *Hd3a* and *OsMADS14*, was upregulated in OE-*PeMADS2* transgenic rice [41]. Another flower promoter’s transcript level (*OsDof12*) also significantly increased in OE-*PeMADS2* lines. *OsDof12* might regulate flowering time by controlling the transcription levels of *Hd3a* and *OsMADS14* independent of other flowering genes under LD [42]. *PheDof12-1* is the homologous gene of *OsDof12* in Moso bamboo; overexpressing *PheDof12-1* in *Arabidopsis* causes early flowering under LD conditions [43]. In contrast, the flowering repressor *DTH8/Ghd8* was repressed in OE-*PeMADS2* lines compared with WT. *DTH8* suppressed rice flowering by down-regulating the expressions of *Ehd1* and *Hd3a* under LD conditions independent of *Hd1* and *Ghd7* [44]. In addition to upstream flowering regulators, rice florigens *Hd3* and *RFT1* were upregulated in OE-*PeMADS2* transgenic rice plants [40]. These two florigens are fundamental for the flowering regulatory pathway because almost all flowering pathways finally target these two genes [45]. Furthermore, two *SUQA* clade MADS-box genes, namely, *OsMADS14* and *15*, were upregulated in OE-*PeMADS2* plants; these are major genes located downstream of *Hd3a* and *RFT1* that regulate the identity of floral meristem development [46]. *PeMADS2* had high homology with *OsMADS18* (*SUQA*-like clade)*,* which can interact with OsMADS15 and OsMADS34 in the shoot apical meristem (SAM) in the reproductive transition [25,34]. Another rice MADS-box gene affected in OE-*PeMADS2* transgenic rice was *OsMADS1,* which was expressed preferentially in flowers and played crucial roles in the determination and specification of floral organ identity in rice [47]. Ectopically expressed *OsMADS1* in tobacco and rice caused early flowering [48]. OsMADS1 could interact with SUQA proteins (OsMADS14 and 15) [49].

These observations suggested that the ectopic expression of *PeMADS2* in rice affected the expressions of upstream genes *OsMADS51, OsDof12,* and *Ghd8*, which were regulated by photoperiod. They either promote or restrain florigen gene expression, enhance the florigen genes *Hd3a, RFT1* expression levels, and ultimately increase the expressions of the downstream genes *OsMADS14, OsMADS15,* and *OsMADS1*, which could control the transformation from SAM to inflorescence meristem (IM) [50]. *Ehd1* is a key flowering time regulator in rice. The expression levels of the upstream and downstream genes involved in the *Ehd1*-dependent pathway showed significant changes (Figure 7A). However, the expression level of *Ehd1* was not notably different between OE-*PeMADS2* and WT plants. One of the assumptions is that PeMADS2 could directly interact with the upstream MADS proteins, OsMADS51/OsMADS65 or OsMADS1, in the regulation of flower development (independent from *Ehd1*) and increase the expression levels of downstream gene *Hd3a/RFT1* and OsMADS14/15. Another assumption is that PeMADS2 could interact with the downstream MADS proteins, such as OsMADS14 and OsMADS15, thereby resulting in feedback regulations that upregulate the expression of upstream genes (*Hd3a/RFT1* and *OsMADS51/OsMADS65*). A similar result was found in *OsMADS15* ectopic expression plants, which was the ultimate downstream target of all the flowering regulators; however, the expression levels of the upstream regulators were upregulated in the transgenic lines [51]. The interactions among different flowering regulators are essential for their function in controlling flowering processes. In this study, *PeMADS2* might be a crucial component of the interaction networks for floral transition in bamboo and could interact with other regulators to determine the flowering time in rice and *Arabidopsis*. The ectopic expression of *PeMADS2* coordinately upregulated the other flowering regulators’ expression levels and might form protein complexes to promote the transition from the vegetative to the reproductive stage.

## 4. Materials and Methods

### 4.1. Plant Materials and Growth Conditions

Flowering bamboo (*P. edulis*) materials were collected in Lingchuan Country, Guilin, China. Wild type and T-DNA-tagged *Arabidopsis* Colombia 0 (Col-0) were grown under long-day photoperiod conditions (16 h/8 h light/dark photoperiod). Rice (*O. sativa*) var. japonica “Dongjing” plants were cultivated in the field of Lin’an, Hangzhou, China.

### 4.2. Gene Cloning and Phylogenetic Analysis

Specific primers were designed to amplify the full-length ORF sequences of bamboo SQUA-like genes (Supplemental Table S1) using cDNA templates prepared from flower bud and leaf tissues. The amplified PCR products were cloned into the pMD18-T vector (Takara, Dalian, China). Five bamboo and related *Oryza* and *Arabidopsis* SQUA-like proteins were aligned by MAFFT version 7 (http://mafft.cbrc.jp/alignment/server/large.html, accessed on 3 October 2021) [52]. A phylogenetic tree was generated using the neighbor-joining method in the MEGA7 program [53,54]. The reliability of tree nodes was evaluated by bootstrap analysis for 1000 replicates.

### 4.3. DGE Data and Transcriptome Data Analysis

*PeSQUAs* expression profiles in bamboo at different inflorescence development stages (F1–F4) and non-flowering bamboo tissues (leaf) were analyzed by quantitative qPCR. For bamboo reproductive transition, the transcriptome data of leaf samples from the following developmental stages were determined: the juvenile stages of 3-week-old seedlings (TW) and 1-year-old plants (OY); the transition stage, i.e., plants that will flower in the next year (FLNY); and the flowering stage, including the flowering plants’ leaves (FL) and flower florets (FP) [30]. Accession number: SRR8053492-SRR8053506. We used HISAT2-2.0.4 (https://daehwankimlab.github.io/hisat2/ accessed on 14 December 2020) to map the reads to the Moso bamboo reference genome (http://www.bamboogdb.org/ accessed on 12 July 2021). StringTie and ballgown were used to estimate the expression levels of all transcripts and to determine the expression level of mRNAs by calculating FPKM [55].

### 4.4. Yeast Two-Hybrid Analysis

Yeast two-hybrid analyses were performed by the Matchmaker^®^ Gold System (Clontech, Palo Alto, CA, USA). The protein-coding sequences of *PeSQUAs* were amplified by PCR using specific primers (Supplementary Table S2). *PeSQUAs* sequences were cloned into both pGBKT7 (bait) and pGADT7 (prey) vectors. Meanwhile, 19 bamboo MADS-box sequences from other subfamilies were also amplified and cloned into pGADT7 vectors separately. The resulting recombinant plasmids were introduced into yeast strains Y2HGold and Y187. Two-hybrid interactions were assayed on selective SD/-Trp/-Leu double-dropout (DDO) and SD/-Trp/-Leu/-His/-Ade/X-α-gal (40 mg/mL) media supplemented with Aureobasidin A (AbA).

### 4.5. Binary Plasmid Construction and Analysis of Transgenic Plants

The ORFs of full-length *PeMADS2* was inserted into the binary vector pCAMBIA1301 using the Cauliflower mosaic virus (CaMV) 35S promoter to drive constitutive expression. Recombinant vectors were transferred to Agrobacterium tumefaciens strain GV3101 and used to transform *Arabidopsis* by the floral dip method [56]. *Arabidopsis* seeds were harvested after transformation and were selected with hygromycin. The flowering times were measured by counting rosette leaf numbers on main inflorescences. The recombinant vector pC1301-*PeMADS2* was also transformed into *O. sativa* “Dongjing” plants. Calli induced from seeds were co-cultured with A. tumefaciens, and the putative transgenic rice was regenerated from calli [57]. The expression levels of *PeMADS2* and flowering regulator genes in transgenic plants were analyzed by qPCR, the gene-specific primers for qPCR are listed in Supplemental Table S3.

### 4.6. RNA Extraction, and Quantitative RT–PCR (qPCR) Analysis

Total cellular RNA was extracted from frozen plant samples using TRIzol reagent kit (Invitrogen, Carlsbad, CA, USA) according to the manufacturer’s protocols. The purity and concentration of total RNA was measured by NanDrop 2000 spectrophotometer (Thermo Fisher, Wilmington, NC, USA) at a wavelength of 230, 260 and 280 nm. The first-strand cDNA was synthesized using the Prime-Script^®^ RT reagent kit (Takara, Dalian, China) according to the manufacturer’s guidelines. The expression analysis of bamboo *PeSQUAs* at different flowering stages was performed by qPCR using SYBR^®^ Premix Ex Taq II (Takara, Dalian, China) on a CFX-96-well Real-Time System (BioRad, Hercules, CA, USA). The relative expression levels of target genes were calculated using the 2^−ΔΔCt^ method by normalizing *NTB* or *TIP41* as the reference gene. The gene-specific primers are listed in Supplementary Table S3. Statistical analysis was conducted by two-tailed Student’s *t*-tests in Microsoft Excel 2011.

### 4.7. RNA-seq, KEGG, and GO Analysis

Leaves from WT and transgenic rice plants were collected when the first panicle appeared. After total RNA extraction according to the 4.6 protocols, sequencing libraries were generated using NEB-Next^®^ Ultra™ RNA Library Prep Kit for Illumina^®^ (NEB, Ipswich, MA, USA). The libraries were sequenced on Illumina Novaseq™ 6000 (LC-Bio Technology CO., Ltd., Hangzhou, China) by following the recommended protocol. We used HISAT2-2.0.4 to map the reads to the Nipponbare reference annotation. The mapped reads of each sample were assembled using StringTie and merged to reconstruct a comprehensive transcriptome using gffcompare software [55]. StringTie and ballgown were used to estimate the expression levels of all transcripts and to determine the expression level of mRNAs by calculating FPKM [58]. The DEGs were selected with fold change >2 or fold change <0.5 and *p*-value < 0.05 by R package DESeq2. The expression levels of these selected DEGs’ expression were confirmed by qPCR. Gene ontology (GO) enrichment analysis of the DEGs was implemented by the GOseq R package [59] and further annotated against the Kyoto Encyclopedia of Genes and Genomes (KEGG) metabolic pathways database [60]. All the RNA-Seq raw data are available in the NCBI Sequence Read Archive (SRA) under the accessions number SRR16020759-SRR16020764.

## 5. Conclusions

Five PeSQUA-like genes were cloned from *P. edulis* and were further grouped into Cluster I (PeMADS2, 13, and 41) and Cluster II (PeMADS3 and 28). Five PeSQUAs had higher transcript levels in the reproductive period than the juvenile phase and had divergent expression patterns at different flowering stages.Y2H showed that three members of cluster I could interact with multiple PeMADS proteins, whereas PeMADS3 and 28 hardly interacted with other proteins. Based on our results, PeSQUA possessed different functions by forming the complicated protein complexes with other MADS-box proteins at different inflorescence development stages. Furthermore, we have chosen *PeMADS2* for functional analysis. The ectopic expression of *PeMADS2* in *Arabidopsis* and rice triggered early flowering, and the alteration of floral organ development was observed in *Arabidopsis*. RNA-seq and qRT–PCR analyses of plants overexpressing *PeMADS2* indicated that *PeMADS2* might integrate multiple flower signaling pathways to trigger early flowering time in rice. Our results provided valuable information on the interaction patterns of PeSQUA and other MADS-box members during *P. edulis* floral development. Moreover, to fully understand the *PeSQUA* subfamily member’s function related to flowering, additional transgenic investigation of other *PeSQUAs* members in *Arabidopsis* and rice should be conducted.

## Figures and Tables

**Table 1:** DEGs related to rice floral pathway in transgenic plants.

Gene ID	Gene Name	Pe2OX vs. WT			
		log2(fc)	Qval	Regulation	Significant
LOC_Os03g11614	*OsMADS1*	3.863916636	1.76 × 10^−2^	up	yes
LOC_Os03g54160	*OsMADS14*	1.223462598	1.10 × 10^−2^	up	yes
LOC_Os07g01820	*OsMADS15*	4.612040145	1.94 × 10^−2^	up	yes
LOC_Os01g69850	*OsMADS51*; *OsMADS65*	3.610909085	9.48 × 10^−6^	up	yes
LOC_Os08g07740	*DTH8*; *Ghd8*;	−1.103164383	6.26 × 10^−2^	down	yes
LOC_Os06g06320	*Hd3a*	3.627964485	3.37 × 10^−6^	up	yes
LOC_Os06g06300	*RFT1*	4.131154855	3.32 × 10^−7^	up	yes
LOC_Os03g07360	*OsDof12*	1.661444261	9.75 × 10^−3^	up	yes

## Data Availability

Not applicable.

## Supplementary Materials

The following are available online at https://www.mdpi.com/article/10.3390/ijms221910868/s1, Table S1: Primers for PeSQUA gene cloning; Table S2: List of primers used for yeast two-hybrid; Table S3: qPCR primers used for transgenic plants; Table S4: Summary of RNA-seq mapping; Table S5: Summary of significant enriched GO terms in OE-PeMADS2; Table S6: Summary of significant enriched GO terms in WT; Table S7: The flowering time-related genes in transgenic and wild type rice; Table S8: In silico identification of putative CArG-box motifs in Arabidopsis genes promoters.
